# A comparative study for ferro particles cloaking and wetting characteristics

**DOI:** 10.1038/s41598-024-66944-8

**Published:** 2024-07-15

**Authors:** Ghassan Hassan, Bekir Sami Yilbas, Abba Abdulhamid Abubakar, Abdullah Al-Sharafi, Hussain Al-Qahtani

**Affiliations:** 1 Mechanical Engineering Department, King Fahd University of Petroleum and Minerals (KFUPM), 31261 Dhahran, Saudi Arabia; 2 IRC for Renewable Energy and Power, King Fahd University of Petroleum and Minerals (KFUPM), 31261 Dhahran, Saudi Arabia; 3 K.A.CARE Energy Research & Innovation Center, 31261 Dhahran, Saudi Arabia; 4 Turkish Japanese University of Science and Technology, Istanbul, Turkey; 5 Interdisciplinary Research Center for Advanced Materials, King Fahd University of Petroleum and Minerals, 31261 Dhahran, Saudi Arabia

**Keywords:** Hydrophobic, Ferroparticles, Water cloaking, Self-cleaning, Nanoscale materials, Soft materials

## Abstract

Ferro hydrophobic particles possess essential properties for controlling the behavior of suspended substances in water. By adjusting the concentration of these particles, the magnetic force within the fluid carrier can be modified, leading to the emergence of distinct flow structures and patterns on the water's surface. This study examines the cloaking phenomenon exhibited by different ferroparticle conditions, employing both experimental and numerical approaches. Under the magnetic influence, hydrophilic particles can attain cloaking velocities of up to 35 mm/s, while hydrophobic particles remain unaffected by the magnetic force, remaining suspended on the water's surface. Hydrophobization of ferroparticles not only decreases their water-cloaking ability but also alters their magnetic properties. The inherent hydrophobic nature of these particles enhances water surface stability, rendering them valuable in various applications, including biomedical and self-cleaning technologies. This research holds particular significance for manipulating suspended particles in water, particularly in biomedical applications like drug delivery and tissue engineering, as well as for advancing self-cleaning technologies.

## Introduction

Ferrofluids are colloidal suspensions of superparamagnetic nanoparticles suspended in a liquid carrier fluid. These fluids exhibit field-induced flow pattern formation on their surfaces^1^, enable droplet self-assembly^2^, and generate micromagnetic convection currents due to their unique magnetic response. The superparamagnetic nanoparticles in ferrofluids are typically composed of ferri/ferromagnetic metals or metal oxides. Despite surface and structural imperfections, the diminutive size of these nanoparticles confers a superparamagnetic behavior to the fluid; each nanoparticle acts as an independent magnetic domain and can alter its magnetization direction under thermal agitation^3^. Consequently, in the absence of an external magnetic field, ferrofluids display no residual magnetization at room temperature, a phenomenon attributed to Neél and Brownian relaxation^4^. Superparamagnetic particles closely resemble ferri- or ferromagnetic materials in terms of their high susceptibility and nonlinear response to magnetic fields. However, they differ because they do not exhibit magnetic hysteresis, a trait shared with paramagnetic materials^1^.

Ferrofluids, while macroscopically non-ferromagnetic, have a unique response to magnetic fields. Although their micro-particles are ferromagnetic, the induced magnetic ordering—when averaged over numerous particles—dissipates once the magnetic field is released. In contrast, paramagnetic materials do not magnetize in the absence of a magnetic field. For instance, iron transitions to a paramagnetic state at 1050 K due to the thermal disruption of its atomic magnetic moments^5^. The paramagnetic nature of ferrofluids arises from the thermal disorder of particle orientation at ambient temperatures and the propensity of magnetic particles in suspension and cluster in pairs or groups, neutralizing their collective magnetic field. The behavior of iron underscores the intrigue and potential of ferromagnetism, leading to inquiries about the feasibility of developing ferromagnetic ferrofluids and understanding their distinctions from paramagnetic variants^5^. To ensure particle integrity and prevent aggregation, driven by forces like van der Waals and magnetic dipole interactions, nanoparticles must be stabilized^6,7^. This stabilization can be realized through electrostatic methods, which charge the particles, or steric methods, where particles are coated with capping agents to induce interparticle repulsion^4^. Surfactants are commonly employed as capping agents, especially in ferrofluids with an organic carrier liquid.

Magnetically stable and controllable liquids offer advantages in a range of mechanical and biological applications. Ferrofluids are commonly used as liquid seals and lubricants, held in position by magnetic fields, and they have potential in the development of pumps, valves, and adaptive photonic systems^8^. Their unique magnetic buoyancy can be harnessed for separation processes, and their anisotropic heat transfer capabilities make them ideal as heat transfer fluids^9^. Recently, there's been a surge in interest in ferrofluids among researchers focusing on microfluidic^10–12^ and biological^13^ domains. Ensuring the consistent performance of these systems often necessitates a comprehensive examination and fine-tuning of the ferrofluid wetting properties.

The surface morphologies of droplets and wetting processes are governed by the minimization of surface energy within a set of boundary constraints. While there has been limited exploration of interfacial tensions in magnetic environments, even fewer studies have delved into this phenomenon with ferrofluids^14^. Often, it's assumed that magnetic fields don't influence interfacial tensions. Yet, these fields can have a notable impact on surface tension levels, particularly when magnetically sensitive surfactants are involved^15^. Kalikmanov's statistical approach considers magnetic dipoles and ferrofluid carrier liquid molecules separately^16^. Evidence suggests that while there isn't a direct magnetic impact on surface tension, dipole interactions can cause an uneven nanoparticle distribution, leading to a minor increase in surface tension as magnetic field strength increases. Zhukov presented a more nuanced model that incorporates the surface tension tensor and field magnetization^17^. This model anticipates a 0.5% relative alteration in the interfacial tension's tangential component when exposed to a 14 kA/m magnetic field aligned with the water-dodecane boundary. A tangential magnetic field amplifies the interfacial tension, however, a perpendicular field diminishes it. External magnetic fields cause a marginal rise in ferrofluid surface tension^17^, and these interfacial tensions play a role in determining field-induced contact angles^18^.

While magnetic fields have the potential to modify interfacial tensions and contact angles, discerning this effect can be intricate due to the overarching deformation of the ferrofluid surface. Contact angles represent the forces acting on a liquid interface. Interestingly, these forces vary based on the specific length scale under examination, and not all are directly tied to the system's inherent wetting behavior^19^. Consequently, an external force might alter the surface and the perceived contact angle without inducing any change in the wetting states. As confirmed through experiments, the microscopic contact angle is consistent with the Young contact angle^20^. While the microscopic contact angle remains stable, the apparent contact angle exhibits variations in the presence of a magnetic field^21^. Given that the magnetic forces exerted on a ferrofluid surpass gravitational forces by several orders of magnitude, the curvature induced by a magnetic field can be significantly more pronounced than that by gravity. Observing ferrofluid contact angles in magnetic fields might impose a finer magnification compared to gravity-based observations^22^. Furthermore, when subjected to a magnetic field, a sessile ferrofluid droplet undergoes elongation, leading to a reduction in its contact angle^23^. Altering the apparent contact angle doesn't always result in a transformation in wetting attributes. The modification in droplet shape could simply mean that the contact angle remains within its hysteresis range. In some recent experiments, positioning a permanent magnet beneath the substrate caused a ferrofluid droplet to spread out while reducing its contact angle from 70° to 50°^24^. This observation raises questions: it could point to a magnetic field-driven alteration in inherent wetting properties or perhaps suggest that the magnification used to measure contact angles was insufficient.

This study focuses on investigating the cloaking phenomenon exhibited by different ferroparticle conditions under magnetic influence. This aspect is relatively underexplored in the existing literature, and our research fills this gap by providing valuable insights into the behavior of ferroparticles in water under different wetting conditions. In this study, the cloaking behavior of both hydrophilic and hydrophobic ferroparticles when subjected to magnetic forces is investigated. In this regard, the experimental procedures are closely monitored using a high-speed video recording system, and a tracker program is used for the meticulous analysis of the recorded data, focusing on the assessment of particle wetting and cloaking. Furthermore, we evaluate the impact of ferroparticle concentration and condition on the observed cloaking behavior, while also quantifying the droplet pinning forces arising from both magnetic influence and surface tension. The findings of our study have implications for various fields, including biomedical applications such as drug delivery and tissue engineering, as well as self-cleaning technologies. By understanding the cloaking and wetting characteristics of ferroparticles, we can potentially enhance the efficiency and effectiveness of these applications.

## Experimental

**Figure 1:**
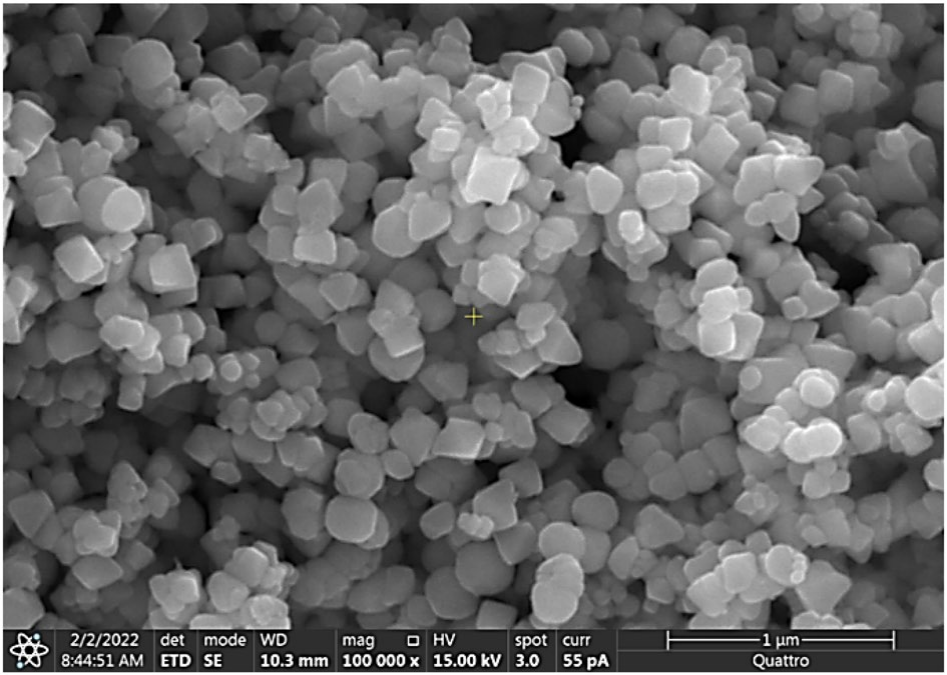
SEM micrograph of the surface created by depositing functionalized silica particles on a crystallized polycarbonate surface.

**Figure 2:**
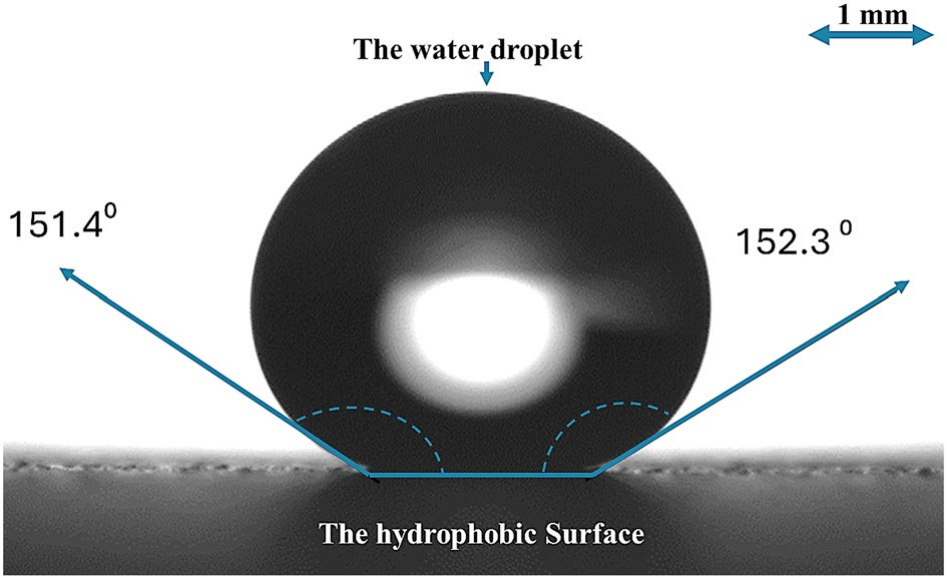
The water contact angle measurement for the textured surface.

Utilizing functionalized silica nanoparticles can alter surface wettability. In previous studies, tetraethyl orthosilicate (TEOS) was employed to functionalize silica particles using the dip-coating method^25^. The initial step in the preparation involved mixing a solution of ethanol (14 mL), ammonium hydroxide (25 mL), and desalinated water (1 mL). This solution was then combined with TEOS (1.5 mL) and isobutytrimethoxysilane (at a 3:4 molar ratio) and stirred for 8 h. The functionalized silica nanoparticles were applied to the glass surfaces (provided by Biolin Scientific) through the dip-coating method. The surface free energy was measured using a goniometer (Data-physics, Model: OCA11), and the microstructure was inspected with a JEOL 6460 SEM. Figure 1 displays the SEM micrograph of the resulting hydrophobic surface of the crystallized polycarbonate surface with texture-functionalized silica particles. The inclusion of silica particles enhances the droplet contact angle and reduces the hysteresis as shown in Fig. 2. A goniometer (Data-physics, Model: OCA11) was used to assess the surface free energy. Due to the addition of silica particles, the droplet contact angle increases to 152° ± 2°, and the hysteresis reduces to 2° ± 1° which results in a hydrophobic surface that enables droplets to roll and slide an inclination of 2° or less.

**Figure 3:**
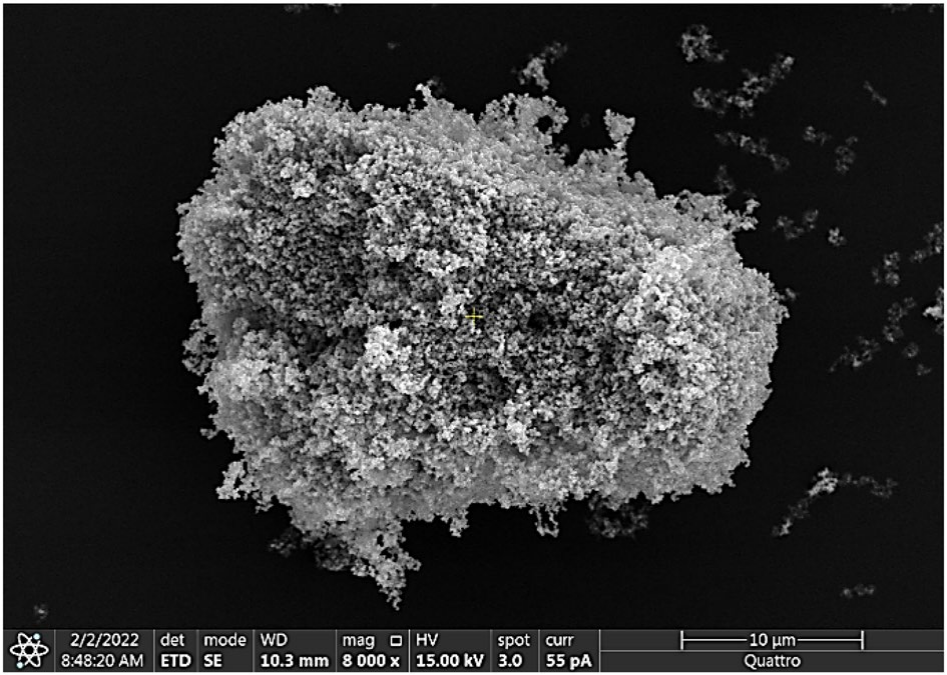
SEM image of the ferro particles functionalized with hydrophobic silica nanoparticles.

In addition, the silica nanoparticles solution was employed to functionalize nanoscale (50 nm) ferroparticles composed of iron (II, III) oxide, Fe3O4, supplied by Sigma Aldrich. Before each droplet experiment, a monolayer of the modified ferroparticles was applied to the water droplet surface. The functionalized silica nanoparticles serve to reduce the surface free energy of the ferro particles, thereby enhancing their hydrophobicity, depending on the specific functional groups attached to the silica nanoparticle coating. By tailoring the composition and properties of the functionalized silica coating, we were able to precisely control the wetting characteristics of the ferro particles, enabling us to investigate their behavior in different liquid environments. Droplets rolling on the hydrophobic glass surface exhibit varied wetting states because of the hydrophobized ferroparticles. Figure 3 displays an SEM image of the ferroparticles functionalized with hydrophobic silica nanoparticles.

**Figure 4:**
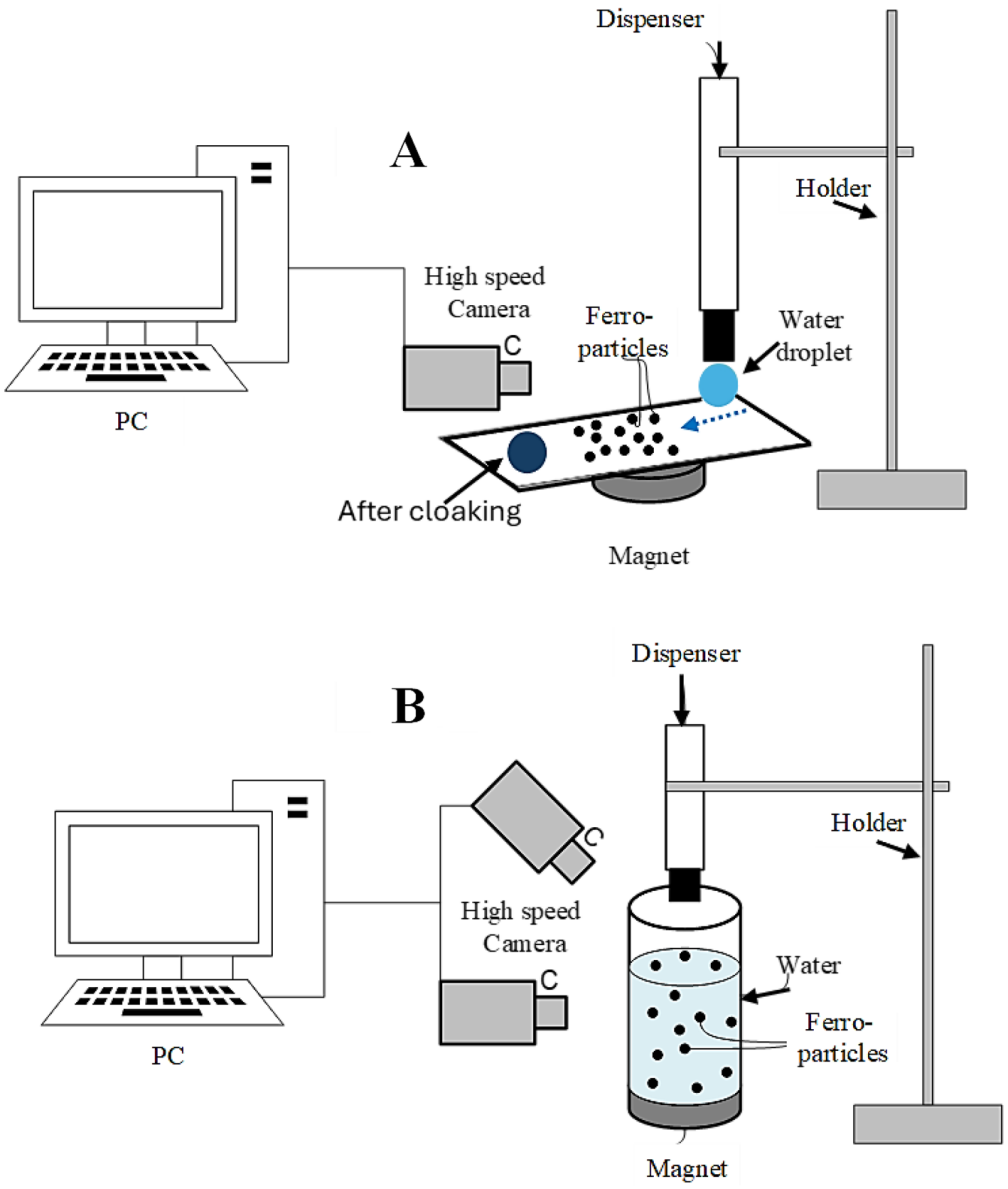
Experimental setup showcasing (A) )ferroparticle cloaking during droplet rolling and (**B**) ferrofluid infusion under the influence of magnetic force.

The studies employ a neodymium iron boron (NdFeB) magnetic core with a maximum magnetic field strength of 1.1806 × 105 A/m, sourced from K&J Magnetics Inc., USA. The fluctuation in magnetic field intensity is measured using a digital tesla meter with a 10 T sensitivity, manufactured by Phywe Systems GmbH & Co. KG. Figure 4 illustrates a typical setup of the experimental apparatus, highlighting the positioning of both the magnet and the modified ferro particles. A micropipette connected to a syringe pump introduces the water droplet. This droplet then navigates through a monolayer of modified ferro particles before being attracted to the magnet. A fixture ensures consistent attraction as the droplet moves over the hydrophobic surface. Droplet dynamics on the hydrophobic plane are captured by a high-speed camera (Speed Sense 9040). Tracker software processes the rapid-motion data. The droplet activities are monitored and recorded at a rate of 5000 frames per second (fps) with a resolution of 1280 × 800 pixels and a pixel size of 14 µm × 14 µm. Data retrieval occurs at 800 fps to bolster data quality in terms of the concentration of data points in a given time frame. According to previous research^26^, uncertainty was assessed, and the standard uncertainty was established at 2%.

## Results and discussion

### Ferrofluid shape in the magnetic field

The theoretical framework presented in the ferrofluid shape in the magnetic field section, serves as a foundation for understanding the behavior of ferrofluids under magnetic influence. Specifically, the concept of magnetization based on fluid continuity is central to our understanding of how ferroparticles respond to magnetic forces and how these forces influence the overall shape and behavior of the ferrofluid. Although our study primarily investigates the behavior of ferroparticles and their interactions, the theoretical concepts presented in the "ferrofluid shape in the magnetic field" section provide valuable insights into the underlying mechanisms governing particle behavior in a magnetic field.

The capacity to control both liquid flow and surface morphology using a magnetic field stands out as one of the most captivating characteristics of ferrofluids. This phenomenon arises from the interchange between the magnetic flux and the dipole moments inherent to each nanoparticle. A magnetic stress tensor aptly captures the relevant forces^1^:

1 $$ {\text{T}}_{{{\text{ij}}}}  =  - \left\{ {\int_{0}^{{\text{H}}} {\upmu _{0} } \left[ {\frac{{\partial {\text{M}}_{\upupsilon } }}{{\partial \upupsilon }}} \right]_{{{\text{H,T}}}} {\text{dH}} + \frac{1}{2}\upmu _{0} {\text{H}}^{2} } \right\}\delta _{{{\text{ij}}}}  + {\text{B}}_{{\text{i}}} {\text{H}}_{{\text{j}}}$$

In this context, H denotes the magnetic field's intensity, T is the temperature, ${\upmu }_{0}$ represents permeability, M signifies fluid magnetization, v is specific volume, ${\updelta }_{\text{ij}}$ is the Kronecker delta function, and B is the magnetic induction. Notably, both magnetic intensity and induction pertain to local, rather than external, field measurements. For more detailed descriptions and derivations, one can express the Magnetic body-force density ${\text{f}}_{\text{m}}$ as follows^27^:

2 $${\text{f}}_{{\text{m}}} = \nabla .{\text{T}} = - \nabla \left\{ {{\upmu }_{0} \mathop \smallint \limits_{0}^{{\text{H}}} \left[ {\frac{{\partial {\text{M}}_{\upsilon } }}{\partial \upsilon }} \right]_{{{\text{H}},{\text{T}}}} {\text{dH}}} \right\} + {\upmu }_{0} {\text{M}}\nabla {\text{H}}$$

In the case of dilute ferrofluids, there is no integral term. However, for concentrated ferrofluids, an integral term exists due to the potential influence of particle density on nanoparticle dipolar interactions. Positioning a permanent magnet with a pronounced field gradient below a surface holding a sessile ferrofluid droplet prompts the droplet to spread out. The last term, directly proportional to the field gradient (h), drives the magnetic material toward regions of enhanced field intensity. Propelled by this force, a ferrofluid droplet mobilizes, enabling the manipulation of non-magnetic objects immersed in the ferrofluid^28^. Similarly, a ferrofluid droplet subjected to a uniform magnetic field tends to stretch in the direction of the field's application. Such deformation amplifies the surface area and, consequently, the energy linked to interfacial tension:

3 $${\text{E}}_{{\text{y}}} = \smallint {\text{A}}\gamma {\text{dA}}$$

The ultimate shape a droplet assumes in equilibrium results from harmonizing the gravitational, magnetic, and surface energies. For smaller droplets, gravitational influences are negligible. The magnetic Bond number $\left({B}_{o}\right)= \frac{2\rho gDH}{\sigma }$ (where D_H_ is the droplet's hydraulic diameter, $\rho$ is the fluid density, g is the gravity and $\sigma$ is surface tension) illustrates the comparative magnitudes of a consistent magnetic field and capillarity^29^.

### Ferro particle cloaking behavior

Liquid infusion on particle surfaces occurs only when the liquid's spreading volume exceeds zero. The spreading coefficient, determined by balancing the solid–vapor surface energy, the liquid–vapor surface tension, and the liquid–solid interface, plays an important role in influencing the rate of liquid spread over solid substrates. The force exerted by surface tension gives rise to the liquid's ability to encompass the particle surface. This act of enveloping a surface with a liquid is termed liquid cloaking. In addition, it has been reported that liquid spreading is proportional to some dimensionless numbers including Reynolds number (Re), Weber number (We), and magnetic Bond number (B_om_)^30^.

**Figure 5:**
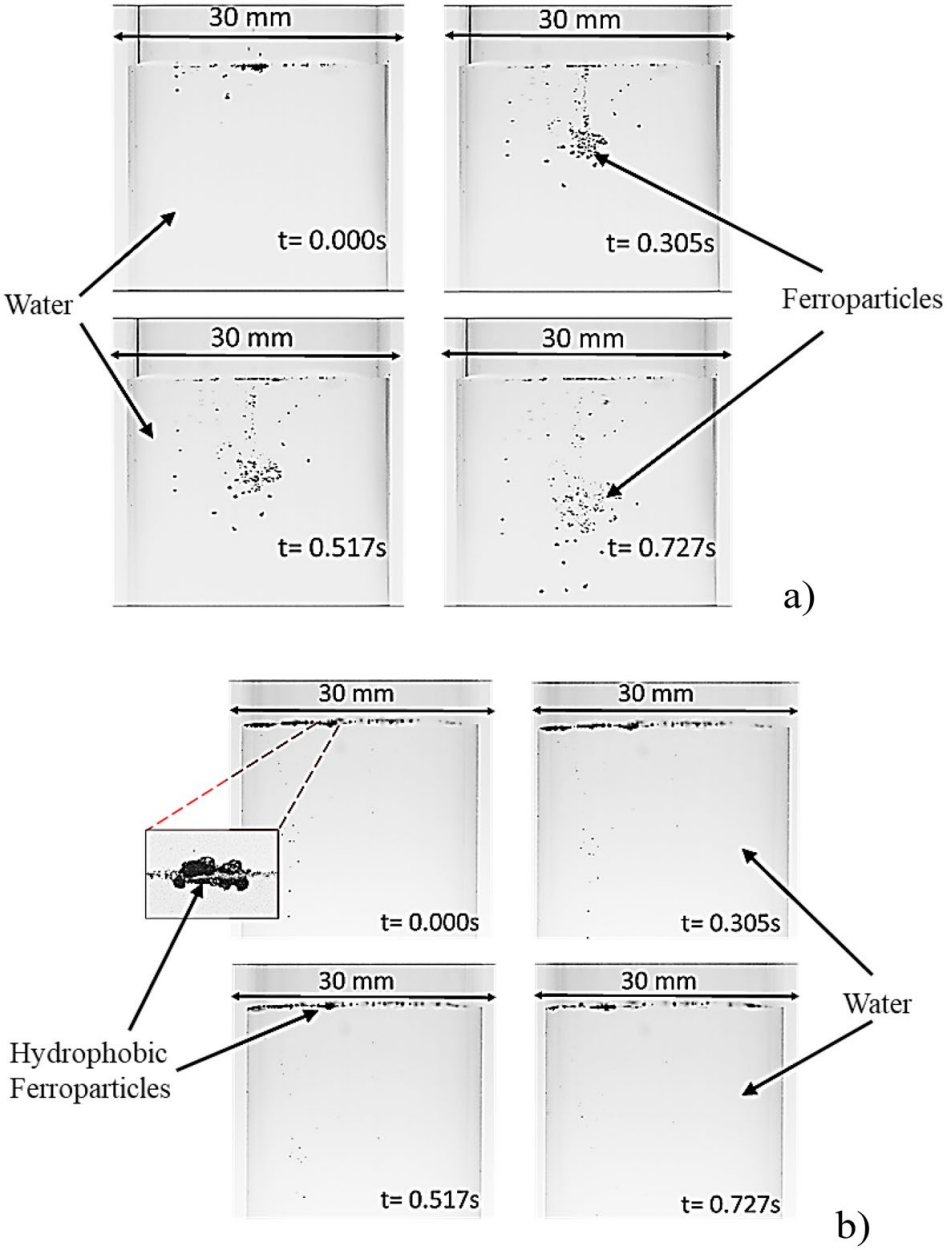
Cloaking behavior of (**a)** Ordinary Ferro particles and (**b)** Hydrophobic Ferro particles.

Comparative tests were performed on both the as-received and the modified Ferro-particles, focusing on the infusion rate of desalinated water (referred to as "cloaking"). Optical images presented in Fig. 4 show the cloaking behaviors of hydrophilic and hydrophobic Ferro-particles immersed in a water medium. During these tests, a high-speed recording device monitored the progression of the liquid ridge's height as the water cloaked the dust particle's surface (Fig. 5a). Subsequently, tracking software quantified the cloaking velocity. Initially, as the liquid is introduced, the liquid ridge on the surface ascends rapidly, but this ascent decelerates once cloaking is initiated. On the other hand, hydrophobic ferroparticles are suspended on the water surface due to their low surface energy and high surface tension of the water (Fig. 5b). The equilibrium between surface tension, the weight of the liquid film, and the resistance from fluid shear governs the rate at which these liquid ridges climb the particle surfaces^31^. The spreading rate (S) is defined as:

4 $$\text{S}={\upgamma }_{\text{s}-\text{a}}-{\upgamma }_{\text{l}-\text{a}}-{\upgamma }_{\text{s}-\text{l}}$$

In this equation, ${\upgamma }_{\text{s}-\text{a}}$ represents the surface free energy, ${\upgamma }_{\text{l}-\text{a}}$ stands for the surface tension, and ${\upgamma }_{\text{s}-\text{l}}$ is the interfacial tension. The droplet contact angle (CA) technique measures the surface free energy of ferroparticles and the fluid^32^.

**Figure 6:**
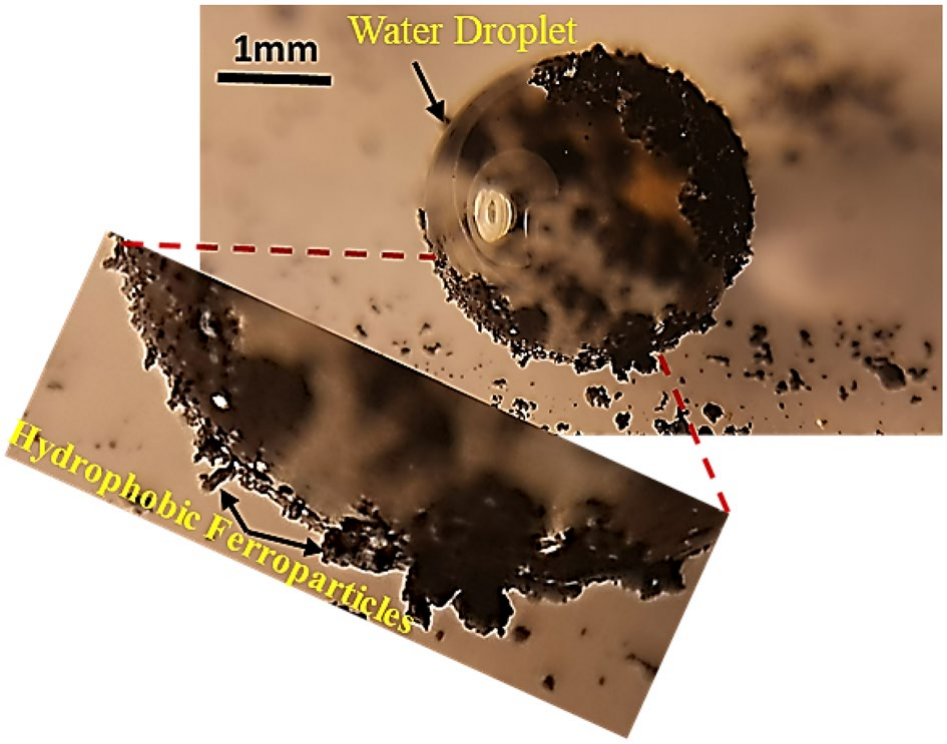
Hydrophobic Ferro-particles attached to the surface of the water droplet.

The surface tension of a liquid is governed by the intermolecular forces between its surface molecules and the contacting substance molecules. When a hydrophobic particle encounters the surface of water, this equilibrium is perturbed. Consequently, the surface molecules can't form a cohesive layer, and the energy that formerly sustained the surface tension prompts the water droplet to reshape, enveloping the particle. This phenomenon is termed the "Bridging Effect". The presence of hydrophobized particles at the sessile drop contact line can significantly alter the wetting characteristics by modifying the local surface energy and interfacial interactions. These particles can affect the spreading and receding dynamics of the droplet, leading to changes in contact angle and droplet morphology. Figure 6 illustrates the attachment of hydrophobic particles to a water droplet's surface. The intensity of the bridging effect can be calculated using the equation:

5 $$\Delta {\text{G}} = 2\gamma \Delta {\text{A}} + \Delta {\text{V}}$$

**Figure 7:**
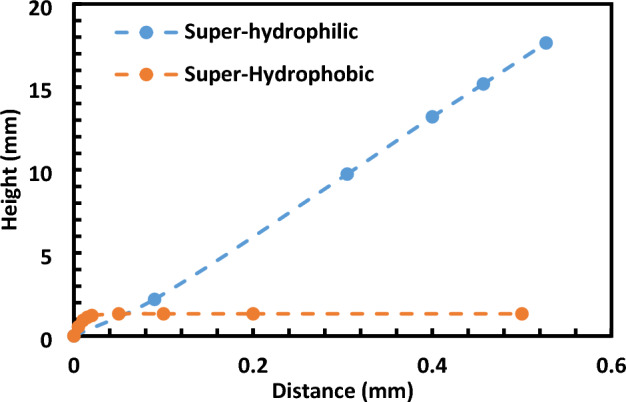
Cloaking height of Ferro-particles. Hydrophobic particles remain suspended at the surface of the water.

Figure 7 contrasts the cloaking heights across various wetting states of the Ferro particles. In this context, ΔG represents the change in free energy, γ is the liquid's surface tension, ΔA denotes the change in surface area, and ΔV is the volume variation of the droplet. When a hydrophobic particle interacts with a water droplet, the surface tension, γ, diminishes due to the particle's influence. Simultaneously, the surface area, ΔA, expands as the droplet deforms to envelop the particle. The volume change, ΔV, is typically insubstantial in this scenario. This interaction culminates in a reduction in the free energy ΔG, rendering the configuration of the droplet and particle energetically preferable. Consequently, hydrophobic ferroparticles will exclusively adhere to a water droplet's surface. This attachment is driven by the bridging effect, a result of the diminished surface tension and augmented surface area induced by the particle's presence. Contrarily, hydrophilic particles get completely cloaked and magnetically drawn to the container's base, as illustrated in Fig. 5a.

### Dynamic model and behavior analysis of hydrophobized ferroparticles on the droplet surface

**Figure 8:**
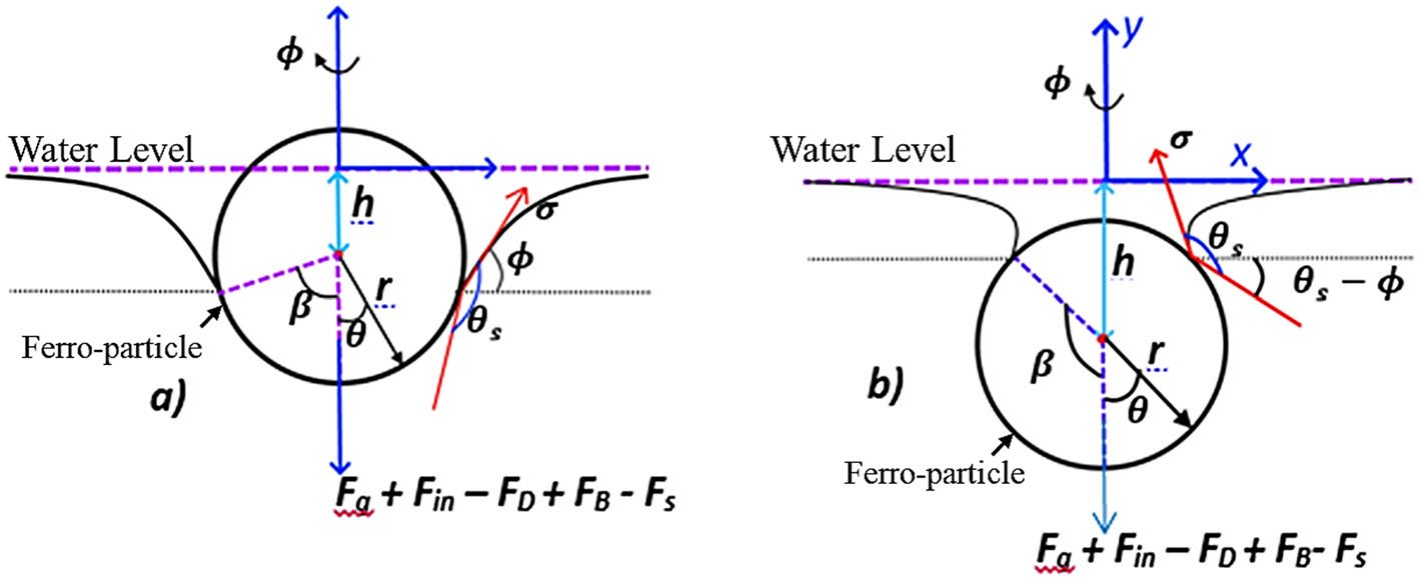
The geometry of hydrophobized Ferroparticle on the top water level.

In the context of hydrophobized ferroparticles and their interaction with rolling droplets, several intricate dynamics come into play, as depicted in Fig. 8. The interplay between the low surface energy of hydrophobized ferroparticles and the surface properties of the droplets becomes particularly evident, especially when the droplets significantly exceed the size of the micron-sized particles. Upon nearing the droplet's surface, a deformative response occurs in the droplet due to the approaching nearly spherical hydrophobized ferroparticle. This interaction initiates a complex interplay of forces, prominently including inertia, surface tension, buoyancy, gravity, and drag. The resultant movement of the particle becomes an outcome of achieving equilibrium among these forces, a delicate balance depicted mathematically within the spherical coordinate system (r, θ, ϕ) and outlined in Eq. (6).

The inclusion of inertia is crucial, particularly as it accounts for the resistance experienced by the particle against changes in its motion state, further influenced by the density and viscosity of the surrounding fluid medium. Surface tension, a fundamental force governing interfacial phenomena, plays a pivotal role, particularly in stabilizing the droplet's shape and influencing the contact angle of the particle with the droplet's surface. Buoyancy and gravity are additional significant factors, with buoyancy buoying the particle upwards due to the density differential between the particle and the surrounding fluid, while gravity tends to pull it downwards. These opposing forces further contribute to the intricate balance dictating the particle's trajectory and interaction with the droplet. Moreover, the drag force, influenced by both the particle's velocity and the fluid properties, acts in opposition to the particle's motion direction, influencing its dynamics as it approaches and interacts with the droplet's surface. Understanding the combined effect of these forces within the spherical coordinate system is essential for predicting the particle's behavior and its subsequent impact on droplet characteristics.

Therefore, the dynamics governing the interaction between hydrophobized ferroparticles and rolling droplets involve a complex interplay of forces, with each force contributing to the particle's trajectory and ultimate integration with the droplet's surface. Considering these dynamics within the theoretical framework outlined by Eq. (6), we gain valuable insights into the intricate physics governing particle-droplet interactions, essential for various applications ranging from surface engineering to microfluidics.

6 $${\text{m}}_{\text{p}}\frac{{\text{d}}^{2}\text{h}}{{\text{dt}}^{2}}=-{\text{F}}_{\text{in}}+{\text{F}}_{\text{s}}+{\text{F}}_{\text{D}}-{\text{F}}_{\text{g}}+{\text{F}}_{\text{B}}$$

Here: ${\text{m}}_{\text{p}}$ is the mass of the ferro-particle, h is vertical displacement into the droplet surface, ${\text{F}}_{\text{in}}$ is inertia force, ${\text{F}}_{\text{s}}$ is surface tension force, ${\text{F}}_{\text{D}}$ is the drag force, ${\text{F}}_{\text{B}}$ is buoyancy force and ${\text{F}}_{\text{g}}$ is the gravitational force.

The surface tension force can be expressed as a function of particle size and contact angle. The surface tension force is quite large for the hydrophobized ferroparticle wetting state, which can result in^33^:

7 $${\text{F}}_{{\text{s}}} = 2\pi {\text{r}}\gamma \;\sin \beta \;\sin \phi$$

Here: $\text{r}$ is particle radius, $\upsigma$ is surface tension coefficient, $\upphi =\uptheta +\upbeta -\uppi ,$ $\uptheta$ is the solid–liquid contact angle, and $\upbeta$ is the angle between the three-phase contact lines.

The gravitational force can be expressed as^33^:

8 $${\text{F}}_{\text{g}}=\frac{4}{3}\uppi {\text{r}}^{3}{\uprho }_{\text{p}}\text{g}$$

Here: ${\uprho }_{\text{p}}$ is particle density, and $\text{g}$ is the gravitational acceleration.

The drag force then becomes:

9 $$ {\text{F}}_{{\text{D}}}  = \frac{{9\uppi }}{{16}}{\text{r}}^{2} \uprho \frac{{{\text{dh}}}}{{{\text{dt}}}}{\text{sin}}^{4} \upbeta  $$

Here: $\upmu$ is air dynamic viscosity. The buoyancy force is:

10 $${\text{F}}_{{\text{B}}} = \frac{\pi }{3}{\text{r}}^{2} \rho {\text{g}}\left( {3{\text{h}}\;\cos^{2} {\upbeta } - 2{\text{R}}\cos^{3} \beta - 3{\text{h}} + 2{\text{r}}} \right)$$

Here: $\uprho$ is the fluid density.

The inertia force becomes:

11 $${\text{F}}_{{{\text{in}}}} = \frac{\pi }{6}{\text{r}}^{3} \rho {\ddot{\text{h}}}\left( {{\text{cos}}^{3} \beta - 3\cos \beta + 2} \right)$$

The force balance equation becomes:

12 $${\text{m}}_{{\text{p}}} {\ddot{\text{h}}} + \frac{\pi }{6}{\text{r}}^{3} \rho {\ddot{\text{h}}}\left( {\cos^{3} \beta - 3\cos \beta + 2} \right) - 2\pi {\text{r}}\sigma \;{\text{sin}}\beta \;\sin \phi - \frac{9\pi }{{16}}{\text{r}}^{2} \rho \frac{{{\text{dh}}}}{{{\text{dt}}}}\sin^{4} \beta + \frac{4}{3}\pi {\text{r}}^{3} \rho_{{\text{p}}} {\text{g}} - \frac{\pi }{3}{\text{r}}^{2} \rho {\text{g}}\left( {3{\text{h}}\;\cos^{2} \beta - 2{\text{R}}\cos^{3} \beta - 3{\text{h}} + 2{\text{r}}} \right) = 0$$

Equation (12) can be solved numerically when $\upbeta$ and $\upphi$ are determined from the experiments using the numerical code developed at home while adopting the initial conditions below:

13 $$\text{h}\left(0\right)=0$$

14 $$\dot{\text{h}}\left(0\right)=0$$

**Figure 9:**
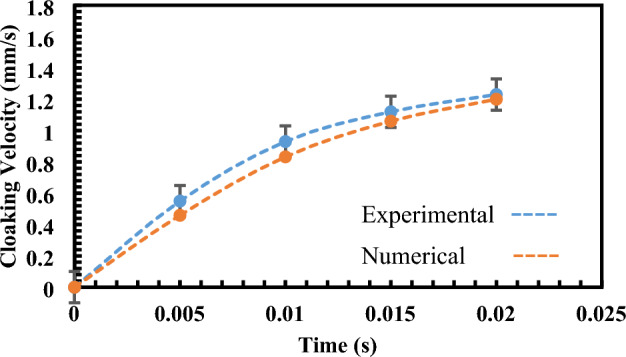
Cloaking velocity of hydrophobic ferroparticles (Experimental and numerical analysis).

As depicted in Fig. 9, the general trajectories of both experimental and numerical data sets align well within the specified time interval. While minor deviations are observable, these discrepancies can be attributed to various factors, including inherent simplifications in numerical modeling, potential experimental errors, and uncertainties in measurement precision and environmental conditions, as previously discussed. The experimental procedure involved repeating the experiment seven times under each condition to ensure robustness and reliability. We meticulously conducted these repetitions to minimize experimental variability and assess the consistency of our results. Through this rigorous approach, we found that the error did not exceed 2% across all repetitions, indicating a high level of precision and reproducibility in our measurements.

### Expression for the falling velocity of ferroparticle below the water surface

The force balance about the falling Ferro-particle results in its acceleration down the water height, which can be expressed as:

15 $$ {\text{E}}_{{\mathrm{p}}} {\text{a}}_{{\mathrm{p}}}  =  - {\text{F}}_{{\mathrm{B}}}  - {\text{F}}_{{\mathrm{D}}}  + {\text{F}}_{{\mathrm{g}}}  p$$

Here: ${\text{a}}_{\text{p}}$ is the Ferro-particle acceleration, ${\text{m}}_{\text{p}}=\frac{4}{3}\uppi {\text{r}}^{3}{\uprho }_{\text{p}}$ is Ferro-particle mass, $\text{r}$ is Ferro-particle radius, ${\uprho }_{\text{b}}$ is particle density, ${\text{F}}_{{\text{B}}} = \frac{4}{3}{\pi r}^{3} \rho {\text{g}}$ is buoyancy force, $\uprho$ is water density, $\text{g}$ is the acceleration due to gravity, ${\text{F}}_{{\text{D}}} = 4\pi \mu {\text{rv}}_{{\text{p}}}$ is the viscous drag force, $\upmu$ is dynamic viscosity, ${\text{v}}_{\text{p}}$ is the particle velocity, and ${\text{F}}_{\text{g}}=\frac{4}{3}\uppi {\text{r}}^{3}{\uprho }_{\text{p}}\text{g}$ is the gravitational force. The Reynolds number of the particle remains low during its transition inside the water; hence the Stokes' flow formulation can be adopted by estimating the drag force. The inertia force on the transiting ferroparticle can be expressed as:

16 $$\frac{4}{3}\pi {\text{r}}^{3} \rho_{{\text{p}}} {\text{a}}_{{\text{p}}} = - \frac{4}{3}\pi {\text{r}}^{3} \rho {\text{g}} - 4\pi \mu {\text{rv}}_{{\text{p}}} + \frac{4}{3}\pi {\text{r}}^{3} \rho_{{\text{p}}} {\text{g}}$$

The rearrangement of Eq. (15) yields:

17 $$\frac{{{\varvec{\rho}}_{{\text{p}}} }}{{\varvec{\rho}}} \cdot \frac{{{\text{a}}_{{\text{p}}} }}{{\text{g}}} = - 1 + \frac{{{\varvec{\rho}}_{{\text{p}}} }}{{\varvec{\rho}}} - \frac{{12\user2{\pi \mu }{\text{R}}}}{{4\user2{\pi \rho }{\text{r}}^{2} \sqrt {{\text{gr}}} }} \cdot \frac{{{\text{v}}_{{\text{p}}} }}{{\sqrt {{\text{gr}}} }}$$

Or

18 $${\text{a}}_{\text{p}}=\frac{-1+\uprho }{\uprho }-\frac{3\uppi }{{\uprho }_{\text{p}}\sqrt{{\text{gr}}^{3}}}\text{v}$$

Here: $\uprho =\frac{{\uprho }_{\text{p}}}{\uprho }$, $\text{a}=\frac{{\text{a}}_{\text{p}}}{\text{g}}$ and $\text{v}=\frac{{\text{v}}_{\text{p}}}{\sqrt{\text{gr}}}$ are the normalized quantities.

The solution to Eq. (17) can be obtained by incorporating the integrating factor, i.e. $\text{exp}\left(\int \frac{3\upmu }{{\uprho }_{\text{p}}\sqrt{{\text{gr}}^{3}}}\text{dt}\right)$. The solution of Eq. (18) yields:

19 $$\text{v}=\frac{{\uprho }_{\text{p}\sqrt{{\text{gr}}^{3}}}}{3\upmu }\left(\frac{-1+\uprho }{\uprho }\right)+\text{Cexp}\left(\frac{3\upmu }{{\uprho }_{\text{p}}\sqrt{{\text{gr}}^{3}}}\text{t}\right)$$

On incorporating the boundary conditions of $\text{v}=0$ at $\text{t}=0$, the constant $\text{C}=-\frac{{\uprho }_{\text{p}\sqrt{{\text{gr}}^{3}}}}{3\upmu }\left(\frac{-1+\uprho }{\uprho }\right)$. Hence, the normalized velocity becomes:

20 $$\text{v}=\frac{{\uprho }_{\text{p}\sqrt{{\text{gr}}^{3}}}}{3\upmu }\left(\frac{-1+\uprho }{\uprho }\right)\left(1-\text{exp}\left(-\frac{3\upmu }{{\uprho }_{\text{p}}\sqrt{{\text{gr}}^{3}}}\text{t}\right)\right)$$

**Figure 10:**
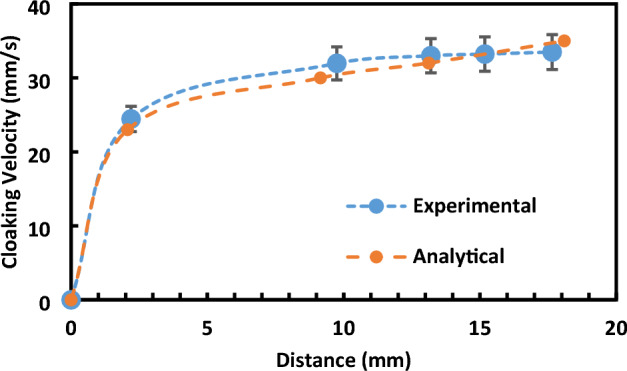
Cloaking velocity of hydrophilic Ferro-Particles (Experimental and analytical analysis).

The velocity profiles showcased in Fig. 10 offer an analytical representation juxtaposed with experimental data, highlighting different cloaking behaviors of ferroparticles based on their inherent wetting states. Intriguingly, the hydrophilic particles, as shown in Fig. 10, exhibit a markedly elevated cloaking velocity—hovering around 30 mm/s—in stark contrast to their hydrophobic counterparts. The essence of this is the natural propensity of hydrophilic particles to gravitate towards water, an attribute that makes them bind and encapsulate it effectively.

Furthermore, it's essential to note that our experimental methodology incorporated rigorous uncertainty analysis and repeatability checks. The experiment was repeated seven times under each condition, ensuring the reliability and robustness of our results. Through this meticulous approach, we maintained a maximum error of 2%, indicating a high level of precision and reproducibility in our measurements. It's particularly noteworthy that the maximum cloaking velocity observed for hydrophilic particles reached around 35 mm/s, which is approximately 30 times higher than the cloaking velocity exhibited by hydrophobic particles Fig. 9. This significant difference underscores the distinct wetting behaviors of hydrophilic and hydrophobic particles and their profound impact on cloaking dynamics.

This potent liaison between hydrophilic particles and water ensures a dominant adhesion, enabling these particles to form intimate connections with myriad surfaces. Their exceptional surface energy is conducive to the wetting of diverse substrates. Moreover, these particles seamlessly integrate into the water, underpinning their easy assimilation and equitable distribution in aqueous configurations. Such nuanced wetting traits of hydrophilic and hydrophobic particles accentuate their adaptability, rendering them indispensable in realms spanning from coatings and adhesives to advanced applications like drug delivery mechanisms.

## Conclusion

The synergistic combination of magnetic and hydrophobic properties in treated hydrophobic ferroparticles presents unique capabilities for manipulating suspended substances in aqueous solutions. The influence of magnetic effects on ferrofluid cloaking is significantly governed by the wetting characteristics of these particles. By adjusting the concentration of ferroparticles, internal magnetic forces can be finely tuned, resulting in the formation of distinct patterns on the water's surface. Through a comprehensive analysis encompassing both experimental and numerical methodologies, the cloaking velocity of various ferroparticle conditions has been thoroughly investigated. Remarkably, hydrophilic particles demonstrate rapid cloaking speeds of up to 35 mm/s, while hydrophobic particles predominantly reside on the water's surface. The application of a hydrophobic coating not only impedes water cloaking around the ferroparticles but also alters their intrinsic magnetic behavior. Moreover, the hydrophobic attributes enhance the stability of the water's surface by repelling water droplets, thereby reducing the total water–air interface area. Consequently, hydrophobic ferroparticles exhibit substantial promise across a wide array of applications, ranging from water purification and oil spill remediation to precise drug delivery systems.

## Data Availability

All data generated or analyzed during this study are included in this published article (and its Supplementary Information files).
